# The mechanism of H171T resistance reveals the importance of N_δ_-protonated His171 for the binding of allosteric inhibitor BI-D to HIV-1 integrase

**DOI:** 10.1186/s12977-014-0100-1

**Published:** 2014-11-25

**Authors:** Alison Slaughter, Kellie A Jurado, Nanjie Deng, Lei Feng, Jacques J Kessl, Nikoloz Shkriabai, Ross C Larue, Hind J Fadel, Pratiq A Patel, Nivedita Jena, James R Fuchs, Eric Poeschla, Ronald M Levy, Alan Engelman, Mamuka Kvaratskhelia

**Affiliations:** Center for Retrovirus Research and Comprehensive Cancer Center, College of Pharmacy, The Ohio State University, 496 W. 12th Ave, 508 Riffe Building, Columbus, OH 43210 USA; Department of Cancer Immunology and AIDS, Dana-Farber Cancer Institute and Department of Medicine, Harvard Medical School, Boston, MA 02215 USA; Department of Chemistry and Center for Biophysics and Computational Biology, College of Science and Technology, Temple University, Philadelphia, PA 19122 USA; Department of Molecular Medicine & Division of Infectious Diseases, Mayo Clinic College of Medicine, Rochester, MN 55905 USA; Division of Medicinal Chemistry and Pharmacognosy, College of Pharmacy, The Ohio State University, Columbus, OH 43210 USA

**Keywords:** HIV-1 integrase, Allosteric inhibitors, Aberrant multimerization, Drug resistance

## Abstract

**Background:**

Allosteric HIV-1 integrase (IN) inhibitors (ALLINIs) are an important new class of anti-HIV-1 agents. ALLINIs bind at the IN catalytic core domain (CCD) dimer interface occupying the principal binding pocket of its cellular cofactor LEDGF/p75. Consequently, ALLINIs inhibit HIV-1 IN interaction with LEDGF/p75 as well as promote aberrant IN multimerization. Selection of viral strains emerging under the inhibitor pressure has revealed mutations at the IN dimer interface near the inhibitor binding site.

**Results:**

We have investigated the effects of one of the most prevalent substitutions, H171T IN, selected under increasing pressure of ALLINI BI-D. Virus containing the H171T IN substitution exhibited an ~68-fold resistance to BI-D treatment in infected cells. These results correlated with ~84-fold reduced affinity for BI-D binding to recombinant H171T IN CCD protein compared to its wild type (WT) counterpart. However, the H171T IN substitution only modestly affected IN-LEDGF/p75 binding and allowed HIV-1 containing this substitution to replicate at near WT levels. The x-ray crystal structures of BI-D binding to WT and H171T IN CCD dimers coupled with binding free energy calculations revealed the importance of the N_δ_- protonated imidazole group of His171 for hydrogen bonding to the BI-D *tert*-butoxy ether oxygen and establishing electrostatic interactions with the inhibitor carboxylic acid, whereas these interactions were compromised upon substitution to Thr171.

**Conclusions:**

Our findings reveal a distinct mechanism of resistance for the H171T IN mutation to ALLINI BI-D and indicate a previously undescribed role of the His171 side chain for binding the inhibitor.

**Electronic supplementary material:**

The online version of this article (doi:10.1186/s12977-014-0100-1) contains supplementary material, which is available to authorized users.

## Background

Rapid evolution of HIV-1 phenotypes conferring resistance to current antiretroviral therapies is a major clinical problem. The multifunctional nature of HIV-1 integrase (IN) provides attractive and unexploited targets for developing complementary antiretroviral compounds to enhance the treatment options for HIV-1 infected patients. During the early stage of HIV-1 replication, IN mediates integration of the reverse transcribed viral genome into human chromatin. This activity proceeds in two steps with the first step, termed 3’ processing, occurring when IN cleaves a GT dinucleotide from the 3’ ends of the viral DNA. The second step, a transesterification reaction termed strand transfer, inserts the processed viral DNA ends into host chromosomal DNA [1]. Three clinically approved antiretroviral drugs raltegravir (RAL), elvitegravir (EVG) and dolutegravir (DTG) inhibit IN strand transfer activity and are collectively referred to as IN strand transfer inhibitors or INSTIs [2]. Importantly, HIV-1 mutations that confer cross-resistance to both RAL and EVG have been identified in patients [3-5]. While the second generation INSTI, DTG, appears to exhibit a higher genetic barrier to resistance, substitutions in IN that confer low-level resistance to DTG have been identified [6].

IN catalytic activities depend on the correct assembly of the stable synaptic complex (SSC) or intasome, where individual IN subunits engage the viral DNA ends to form the fully functional IN tetramer [7]. Each of the three IN domains, the N-terminal domain (NTD), the catalytic core domain (CCD) and the C-terminal domain (CTD), contribute to the assembly of the SSC through protein-protein and protein-DNA interactions [8-12]. Unliganded IN subunits exhibit highly dynamic interplay with the inhibition of this exchange through the stabilization of subunit-subunit interactions prior to their binding to viral DNA results in the loss of enzymatic function [11,13]. Initial studies with the small molecule inhibitor tetra-acetylated-chicoric acid have shown that the inhibitor binds at the IN dimer interface and promotes the incorrect multimerization of IN, which in turn compromises IN catalytic activity *in vitro* [14]. These findings have provided important proof-of-concept for a new mechanism for inhibition of IN activity through the modulation of its multimeric state.

Integration in infected cells is significantly enhanced by the cellular chromatin associated protein LEDGF/p75 which acts as a bimodal tether to link the lentiviral preintegration complex to active genes [15-20]. LEDGF/p75 association with chromatin is mediated through its N-terminal segment containing the PWWP domain, which selectively recognizes the H3K36me3 histone mark as well as non-specifically engages nucleosomal DNA [21-23]. LEDGF/p75 also binds the IN tetramer through its C-terminal integrase binding domain (IBD) by inserting a small loop into a V-shaped cavity located at the HIV-1 IN CCD dimer interface [20,24-26]. LEDGF/p75 Asp366 establishes a pair of hydrogen bonds with IN Glu170 and His171 backbone amides, whereas LEDGF/p75 Ile365 and Leu368 engage in hydrophobic interactions with both IN subunits [20,24]. In addition, the LEDGF/IBD α-helix 4 forms electrostatic interactions with α-helix 1 of the IN NTD [26]. Antagonism of HIV-1 IN interaction with LEDGF/p75 through knockout (KO) of the cellular *Psip1* gene, which encodes for LEDGF/p75 protein, resulted in marked decrease of HIV-1 infectivity [18,27,28]. Additionally, overexpression of the LEDGF/IBD, which is capable of both competing with endogenous LEDGF/p75 as well as inhibiting the formation of the SSC by stabilizing incorrect IN multimers [13], was able to potently inhibit HIV-1 replication [17,29]. These studies have established the importance and molecular basis of the interaction of HIV-1 IN with LEDGF/p75 and have highlighted the primary LEDGF/p75 binding pocket at the IN CCD-CCD dimer interface for anti-HIV-1 drug development.

Multifunctional allosteric IN inhibitors (ALLINIs) have been discovered that potently inhibit HIV-1 replication (reviewed in [30-33]). These compounds were identified through two separate methods, including a high throughput screen for 3’-processing inhibitors or through the rational design of inhibitors that block the IN-LEDGF/p75 interaction [33-36]. ALLINIs bind to HIV-1 IN in the principal LEDGF/p75 binding pocket and bridge between two IN subunits [36-42]. ALLINIs share several common structural features including a central quinoline ring and the carboxylic acid moiety on a modifiable one-carbon linker attached to position 3 of the central ring. Similar to LEDGF/p75 residue Asp366, the ALLINI carboxylic acid forms hydrogen bonds with Glu170 and His171 backbone amides of one IN subunit. Additionally, the quinoline ring extends to form hydrophobic contacts with the second IN subunit akin to LEDGF/p75 residue Leu368. However, unlike LEDGF/p75, potent ALLINIs also contain a *tert*-butoxy ether oxygen at the modifiable carbon, which forms an additional hydrogen bond with the side chain of IN residue Thr174 [36,37,39,40].

ALLINIs inhibited both IN-LEDGF/p75 binding and LEDGF/p75 independent assembly of functional SSCs *in vitro* [36,37,40,43]. The latter inhibitory activity has been attributed to the ability of ALLINIs to prematurely stabilize interacting IN subunits and promote aberrant higher order protein multimerization [37,40,43]. Consistent with these observations, in infected cells ALLINIs impaired a step at or prior to 3’-processing and could reduce LEDGF/p75 mediated integration into active transcription units [40,44]. Unexpectedly though, the primary activity of ALLINIs occurs during the late stage of HIV-1 replication [39,41,42,45,46]. Virions produced in the presence of ALLINIs exhibited an eccentric morphology characterized by the electron dense material being mislocalized outside of the capsid core and were furthermore defective for reverse transcription during the subsequent round of infection [39,45,46]. This phenotype is similar to the one caused by certain HIV-1 IN mutations, which are typed as class II, suggesting that IN structure may play a yet unidentified role during HIV-1 maturation [12,39,47-51]. Potential contributions of LEDGF/p75 and its interactions with IN during the late stage of HIV-1 replication are unlikely due to the observations that fully infectious virus particles were formed in LEDGF/p75 KO or knockdown cells [27,39,45]. Consistent with this view, LEDGF/p75 over expression did not affect ALLINI potencies in virus producer cells [45]. Instead, ALLINI induced aberrant IN multimerization has been shown to correlate with the inhibition of correct particle assembly. Further support for this notion has been provided by the recent development of multimerization selective IN inhibitors or MINIs. These compounds are not effective inhibitors of IN-LEDGF/p75 binding and instead potently induce aberrant IN multimerization during virus particle production and result in eccentric, non-infectious particles [44].

Genotyping HIV-1 in cell culture under the selective pressure of the archetypal ALLINI BI-1001 and its several analogs have identified substitutions in the IN coding sequence near the inhibitor binding sites [36,40,52]. Of these, the A128T IN substitution was the most prevalent mutation with HIV-1_NL4-3(A128T IN)_ displaying marked resistance to respective ALLINI compounds [36,38,40,52]. Interestingly, crystallographic studies have revealed that BI-1001 is still able to bind A128T IN CCD by maintaining all hydrogen bonding interactions, but that the quinoline ring bridging the two IN subunits was slightly shifted compared with the wild type (WT) protein [38]. Consequently, BI-1001 was unable to promote aberrant multimerization of recombinant A128T IN, whereas it maintained its ability to inhibit IN-LEDGF/p75 binding *in vitro* [38]. The results of these studies are consistent with the interpretation that aberrant IN multimerization rather than IN-LEDGF/p75 binding is the primary target of this inhibitor in infected cells.

Selection of HIV-1 variants in the presence of ALLINI BI-D (Additional file 1: Figure S1), a more potent analog of BI-1001, did not result in the A128T mutation but instead revealed several amino acid changes near the inhibitor binding sites including Y99H, L102F, A/T124D, and H171T [52]. Of these, HIV-1 bearing the single amino acid H171T substitution was found to be one of the most prominent mutations persisting at the highest concentration of BI-D tested [52]. Here, we have investigated the mechanism of resistance for the H171T IN mutation. Our findings show that unlike A128T, the H171T IN substitution causes resistance to BI-D by reducing the binding affinity of the inhibitor to IN. Our structural studies have elucidated a previously undescribed role of the His171 side chain for hydrogen bonding with BI-D *tert*-butoxy ether oxygen, which is compromised upon the H171T substitution. Since LEDGF/p75 lacks a *tert*-butoxy moiety, the H171T substitution has minimal effects on IN-LEDGF/p75 binding and accordingly, HIV-1_NL4-3(H171T IN)_, replicated in cells at WT levels. These findings have uncovered the structural and mechanistic basis for H171T IN resistance to BI-D and are expected to facilitate in the development of second generation ALLINIs with increased potency and decreased potential to evolve drug resistance.

## Results

**Figure 1:**
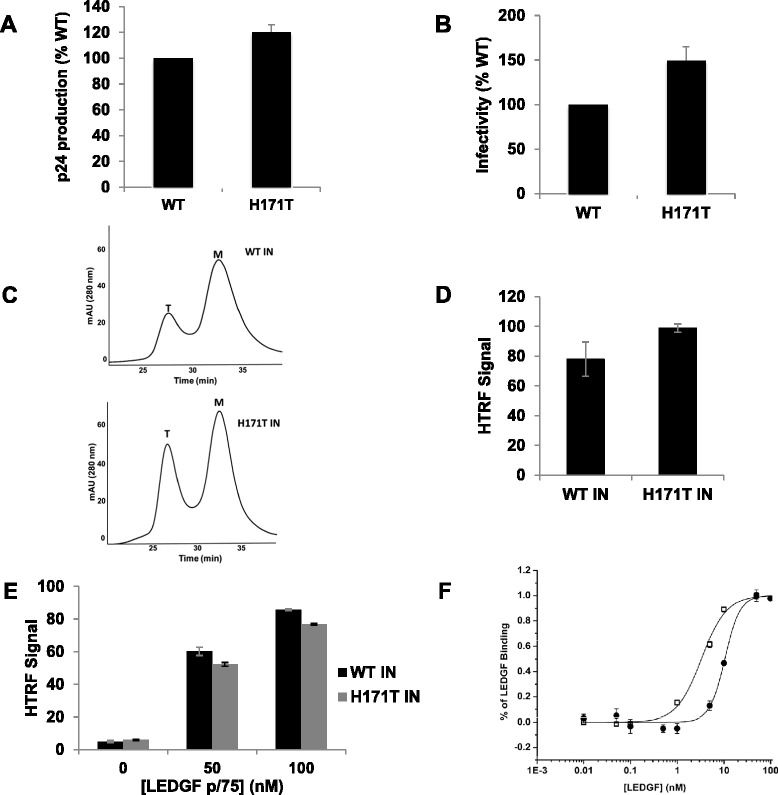
**Effects of the H171T IN substitution on HIV-1 replication and recombinant IN activities. (A)** p24 production of HIV-1_NL4-3_ and HIV-1_NL4-3(H171T IN)_ plotted as percent WT with standard deviations shown for n = 3 independent experiments. **(B)** Single round infection of HIV-1_NL4-3_ and HIV-1_NL4-3(H171T IN)_ determined by luciferase expression and plotted as percent WT infectivity with standard deviations shown for three independent experiments. **(C)** SEC of WT and H171T INs. The peaks corresponding to tetrameric (T) and monomeric (M) forms of IN are indicated. **(D)** HTRF-based LEDGF/p75 independent integration assay and **(E)** HTRF-based LEDGF/p75 dependent integration assays showing stimulation of WT and H171T IN activities at indicated LEDGF/p75 concentrations. Total HTRF signal is plotted with standard deviation for three independent experiments shown. **(F)** HTRF-based assays to determine LEDGF/p75 binding affinities for WT (opened boxes) or H171T (closed circles) INs. Error bars indicate standard deviation for three independent experiments.

To assess the functional significance of the H171T IN substitution we introduced the mutation into both HIV-1_NL4.3_ and recombinant IN. As indicated in Figure 1A and B, the H171T IN substitution did not significantly alter virus release from transfected cells as measured by p24 production or affect the infectivity of the mutant virus. We next examined the biochemical properties of purified recombinant H171T IN. Size exclusion chromatography (SEC) experiments revealed that WT and H171T INs similarly formed tetramers and monomers (Figure 1C). In a homogeneous time-resolved fluorescence-based (HTRF) IN activity assay [38], the mutant IN protein exhibited near WT levels of catalytic function in the absence of LEDGF/p75 (Figure 1D). Furthermore, LEDGF/p75 was able to stimulate the strand transfer activity of mutant IN similarly to WT IN (Figure 1E). This suggested that even though the H171T IN substitution is within the IN-LEDGF/p75 binding pocket, IN retains the ability to effectively bind LEDGF/p75 *in vitro* and during virus infection. To test this directly, we compared LEDGF/p75 binding to WT and H171T INs utilizing a HTRF-based binding assay [38]. WT IN bound LEDGF/p75 with a *K*_*d*_ of 3.3 ± 0.3 nM, whereas H171T IN bound LEDGF/p75 with a *K*_*d*_ of ~10.5 ± 0.3 nM, a 3.2-fold decrease in affinity (Figure 1F).

Although the inhibition of particle maturation determines BI-D potency, the inhibitor displays a second, substantially weaker activity during the early phase of HIV-1 replication ([39], Table 1). To gain insight into the mechanism of BI-D action and the mode of H171T resistance, HIV-1_NL4-3(H171T IN)_ was evaluated during both the early and late stages of the replication lifecycle. When drug exposure was limited to the acute phase of infection, BI-D EC_50_ values increased from 1.17 μM for VSV-G pseudotyped HIV-1_NL4-3_ to 12.4 μM for the VSV-G pseudotyped HIV-1_NL4-3(H171T IN)_, ~11-fold resistance (Table 1). However, the H171T IN substitution resulted in significantly higher levels of resistance (~68-fold) when drug exposure was limited to the late stage of replication. To examine the apparent differences in resistance levels between the early and late stages of replication, anti-viral potencies of BI-D were determined in LEDGF/p75 KO cells. The complete removal of endogenous LEDGF/p75 increased BI-D potency during the acute phase of HIV-1_NL4-3_ infection, but had minimal effect on the inhibitor activity when exposure was limited to the late stage. These results are consistent with other studies and indicate that during the early stage of replication, LEDGF/p75 is able to compete with ALLINIs for binding to IN and hence reduce inhibitor potency [39,53]. However, LEDGF/p75 expression levels did not detectably alter ALLINI potencies during the late stage of viral replication ([27,39,41,42,45,46], also see Table 1). The virus containing the H171T IN substitution still conferred resistance to BI-D in the absence of endogenous LEDGF/p75, with 28- and 45-fold resistance observed in the KO cells during the early and late stages, respectively.

**Table 1:** **Effects of the H171T IN substitution on antiviral activities of BI-D**

	**Producer cells**		**Target cells**		**LEDGF KO producer cells**		**LEDGF KO target cells**	
	**EC**_**50**_ **(μM)**	**Fold change**	**EC**_**50**_ **(μM)**	**Fold change**	**EC**_**50**_ **(μM)**	**Fold change**	**EC**_**50**_ **(μM)**	**Fold change**
WT IN	0.090 ± 0.031^*a*^	--	1.17 ± 0.1^*a*^	--	0.080 ± 0.01	--	0.067 ± 0.02	--
H171T IN	6.11 ± 0.63	67.9x	12.4 ± 0.85	10.6x	2.20 ± 0.64	28x	2.98 ± 0.7	44.5x

Means ± S.D. are shown for at least 3 independent experiments.

^a^Data from Ref [39].

**Figure 2:**
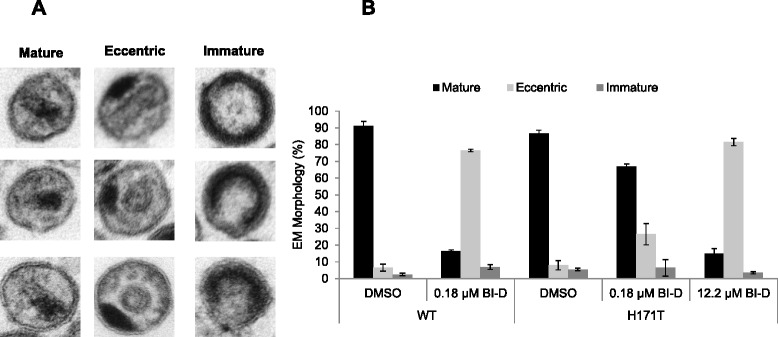
**Concentration dependent effects of BI-D on viral core morphology for HIV-1**_**NL4-3**_ **and HIV-1**_**NL4-3(H171T IN)**_ **. (A)** Representative images of mature, eccentric and immature virion morphologies as visualized by electron microscopy. **(B)** Quantitation of counted virions (100 for WT or H171T per experiment). Virions were produced in the presence of DMSO, 0.18 μM BI-D or 12.2 μM BI-D as indicated. Graphed are averages and standard deviation for n = 2 independent experiments.

The vast majority of virions produced in the presence of BI-D display eccentric core morphology, where the electron dense material normally situated within the conical core is mislocalized adjacent to a translucent capsid core and the viral membrane [39]. Consistent with these findings, when WT HIV-1_NL4-3_ virions were produced in the presence of 0.18 μM BI-D (a dose equivalent to 2×EC_50_; Table 1), 77% of the virions displayed an eccentric morphology (Figure 2). However, at the same concentration of BI-D the H171T IN mutant virus resulted in only 27% of the virions with eccentric core morphologies (Figure 2). When BI-D concentrations were increased to 12 μM, which corresponds to 2×EC_50_ for HIV-1_NL4-3_ bearing the H171T IN substitution, the eccentric virion morphology for the mutant virus increased to 82% (Figure 2).

**Figure 3:**
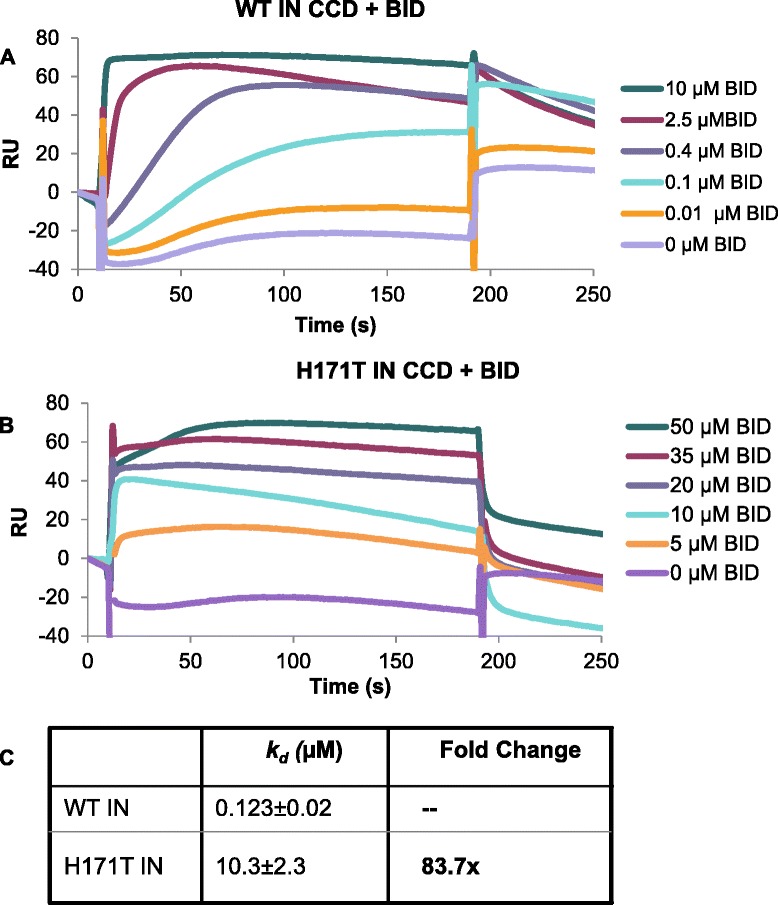
**SPR analysis of BI-D interactions with WT and H171T mutant IN CCDs.** SPR binding kinetics for BI-D interactions with **(A)** WT IN CCD and **(B)** H171T IN CCD at indicated inhibitor concentrations. Binding affinities are summarized in **C**.

We next wished to dissect the mechanism of the HIV-1_NL4-3(H171T IN)_ resistance to BI-D. Surface plasmon resonance (SPR) was used to compare BI-D binding to recombinant WT and H171T IN CCD proteins. Figure 3 shows that BI-D bound WT IN CCD with a *K*_*d*_ of 0.123 μM, which is in good agreement with the antiviral activities of BI-D measured in cell culture (Table 1). BI-D binding to H171T IN CCD resulted in a significantly higher *K*_*d*_ of 10.3 μM. Significantly, the observed ~84-fold decrease in the binding affinity of BI-D to the mutant IN CCD (Figure 3) roughly correlated with the ~68-fold decrease in the EC_50_ value seen for antiviral activity against HIV-1_NL4-3_ bearing the H171T IN substitution (Table 1).

**Figure 4:**
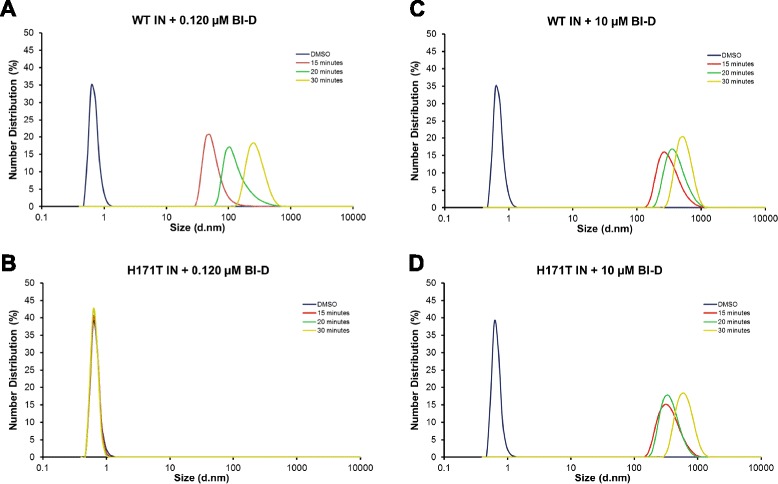
**DLS analysis of BI-D induced oligomerization of recombinant WT and the H171T INs.** Shown are the size distributions (%) of IN after DMSO treatment (blue) or BI-D treatment after 15 minutes (red), 20 minutes (green) and 30 minutes (yellow) incubation. BI-D treatments include **(A)** WT IN +0.120 μM BI-D, **(B)** H171T IN +0.120 μM BI-D, **(C)** WT IN +10 μM BI-D and **(D)** H171T IN +10 μM BI-D. The peak with the diameter size of <1 nm detected in these samples has also been observed for the buffer alone sample indicating that small size particles unrelated to IN or BI-D were present in our preparations.

Previous studies have indicated that the primary mechanism of action of ALLINIs is through the promotion of aberrant IN multimerization [38-41,43]. Therefore, we compared the effects of BI-D on aberrant multimerization of WT and H171T INs using dynamic light scattering (DLS). In the absence of inhibitor (DMSO control), peaks for soluble WT or mutant IN were not observed by this technique (Figure 4). Instead, a background signal corresponding to <1 nm diameter was detected both in the presence of IN and in the buffer alone sample, indicating that the reaction buffer contained small size particles. For inhibitor experiments, we examined two concentrations of BI-D: one (~0.120 μM) that correlated to the *K*_*d*_ value of the inhibitor binding to WT IN CCD (~0.123 μM) and the other (10 μM) that correlated with the *K*_*d*_ value for inhibitor binding to H171T IN CCD (~10.3 μM). Incubation of 0.12 μM BI-D with WT IN for 15 min resulted in a peak corresponding to particles with a diameter of 51 nm, which significantly exceeds an estimated diameter of 7.5 nm for the IN tetramer in the SSC [54]. The size of the oligomer continued to increase further to 106 nm and 142 nm diameter at 20 and 30 minutes, respectively (Figure 4A). In contrast, the same BI-D concentration (0.12 μM) failed to elicit higher order H171T IN oligomers even after 30 min incubation (Figure 4B). However, when the concentration of BI-D was increased to 10 μM, higher order oligomerization of H171T IN was detected in a time dependent manner (Figure 4D). As expected, 10 μM BI-D also induced higher order oligomers of WT IN (Figure 4C). This suggests that at lower concentrations, BI-D is unable to promote higher order IN oligomerization of H171T IN likely due to the decreased affinity of the inhibitor binding to the mutant protein (Figure 3). However, under conditions of increased inhibitor, BI-D is able to bind H171T IN (Figure 3) and promote aberrant IN multimerization (Figure 4D). This indicates that the H171T IN substitution confers resistance to BI-D by decreasing inhibitor binding affinity and hence correspondingly decreasing aberrant IN multimerization.

**Figure 5:**
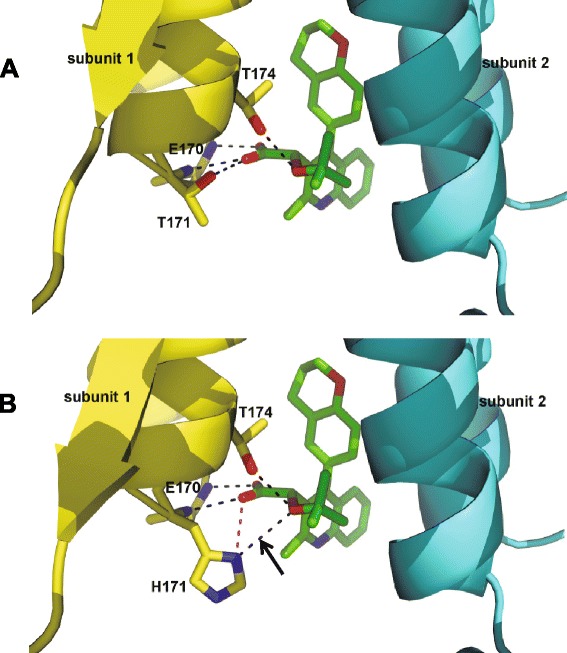
**Crystal structures of BI-D bound to WT and H171T CCD dimers.** Panel A is the H171T CCD dimer and panel B is the WT CCD dimer. BI-D is colored green and individual IN subunits are colored yellow and cyan. Oxygen atoms are shown in red and nitrogen atoms are in blue. Black dash-lines indicate hydrogen bonding interactions, whereas the magenta dash-line shows the electrostatic interaction between the protonated N_δ_- on His171 and the carboxylic acid of BI-D. The arrow indicates the hydrogen bond between the protonated N_δ_- on His171 and the ether oxygen on the *tert*-butoxy **(B)**, which is absent in the H171T IN CCD structure **(A)**.

To understand the structural basis for the reduced binding affinity of BI-D to H171T IN, we solved the crystal structure of BI-D in complex with H171T CCD dimer (Figure 5A) and compared it to the complex of inhibitor bound to WT CCD dimer ([39], also see Figure 5B). As expected (Figure 3) with the high concentration of BI-D (~5 mM) used in the crystallographic experiments, the inhibitor bound to H171T CCD dimers. Furthermore, the H171T substitution did not detectably affect the inhibitor position within the binding pocket (compare Figure 5A and B). BI-D hydrophobic interactions with IN CCD subunit 2 as well as hydrogen bonding between the inhibitor carboxylic acid, and backbone amides of subunit 1 were fully preserved in both crystal structures. Furthermore, the Thr174 side chain similarly hydrogen bonded to the *tert*-butoxy ester oxygen in both the WT and mutant IN structures.

Importantly, though, we observed differential interactions of His171 and Thr171 side chains with the inhibitor. The imidazole group of His171 formed both an electrostatic interaction with the carboxylic acid and a hydrogen bond with the *tert*-butoxy oxygen of BI-D (Figure 5B). However, the side chain of Thr171 which establishes a novel hydrogen bond with the carboxylic acid was unable to form a hydrogen bond with the *tert*-butoxy moiety of BI-D (compare Figure 5A and B).

To understand how these structural differences contributed to the markedly reduced ability of the inhibitor to bind H171T IN, we performed absolute binding free energy calculations for the interactions between WT and H171T IN CCD dimers with BI-D (Table 2 and Additional file 1: Figure S2). N_δ_-, N_ε_- and doubly (N_δ_- and N_ε_-) protonated forms of His171 were considered in our calculations. Table 2 shows that the doubly-protonated form of His171 and the N_δ_-protonated form of His171 have similar calculated ΔG_bind_ (cal) of -11.2 kcal/mol and -10.1 kcal/mol respectively; whereas a significantly weaker ΔG_bind_(cal) value of -5.9 kcal/mol was obtained for the Nε-protonated form of His171. The calculated ΔG_bind_ (cal) for the IN containing Thr171 was -6.7 kcal/mol (Table 2). For comparison, Table 2 also shows the ΔG_bind_ (exp) values determined using experimental *K*_*d*_ values for BI-D binding to WT and H171T IN CCDs (Figure 3). Comparison of ΔG_bind_ (cal) and ΔG_bind_ (exp) suggests that BI-D binding to WT IN preferentially stabilizes the doubly protonated form and/or the N_δ_-protonated tautomer states of His171, while the N_ε_-protonated tautomer is a relatively minor species in the inhibitor-bound complex. We also decomposed the computed binding free energy ΔG_bind_ (cal) into contributions from electrostatic and non-polar interactions. We compared the results for both BI-D binding to the H171T IN CCD and its WT counterpart (data not shown), which suggests that the less favorable electrostatic interactions between BI-D and Thr171 primarily contribute to the lower *K*_*d*_ value for the inhibitor binding to the mutant protein. Significantly, comparisons between the molecular dynamics (MD) simulated structures obtained with different protonation states of His171 and the crystal structure of BI-D in complex with the WT IN CCD dimer are consistent with the trend in the computed binding free energies (see Additional file 1: Table S1 and Figure S2).

**Table 2:** **Binding free energy calculations for BI-D interactions with WT and the H171T mutant IN CCDs**

**Receptor**	**ΔG**_**bind**_ **(cal)**	**ΔG**_**bind**_ **(exp)**
WT IN (His171-doubly-protonated)	-11.2	-9.5
WT IN (His171-Nδ-protonated)	-10.1	
WT IN (His171-Nε-protonated)	-5.9	
His171T IN	-6.7	-6.8

Unit: kcal/mol. ∆G_bind_ (cal) represent calculated values for BI-D binding to His171 containing either Nδ-, Nε- or doubly protonated tautomer states and H171T IN CCDs. ∆G_bind_ (exp) values have been calculated based on the experimental data.

The simulations also revealed the importance of hydrogen bonding interactions between the ether oxygen on the *tert*-butoxy moiety on BI-D and the protonated N_δ_- on His171. In contrast, to the mutant IN CCD, the hydroxyl group of the Thr171 side chain does not form such interactions with the ether oxygen on the inhibitor (Figure 5). This result is supported by the published observations that ALLINIs which contain an oxygen ether linkage are considerably more potent inhibitors of HIV-1_NL4.3_ with WT IN than those lacking such an oxygen atom (reviewed in [31]). To further test this notion we have examined the effects of the H171T IN substitution on the antiviral activities of LEDGIN-6, which lacks the *tert*-butoxy group ([36,37], also see Additional file 1: Figure S1). LEDGIN-6 was only ~3.4-fold less potent with respect to HIV-1_NL4.3 (H171T IN)_ (EC_50_ of 41.4 ± 6.4 μM) (data not shown) versus WT HIV-1_NL4.3_ (EC_50_ of 12.2 ± 2.9 μM) [37]. For comparison, BI-D, which unlike LEDGIN-6 contains the *tert*-butoxy group, was significantly more sensitive (~68-fold, Table 1) to the H171T IN substitution in HIV-1_NL4.3_ likely due to the disruption of the hydrogen bonding between the *tert*-butoxy ether oxygen and N_δ_-protonated His171.

Next, we wanted to understand the structural basis as to why the H171T substitution had significantly less effect on LEDGF/p75 binding (~3.2-fold) compared with BI-D binding (~84-fold) to recombinant HIV-1 IN. Comparison of available crystal structures of BI-D or LEDGF/IBD bound to WT HIV-1 IN CCDs [20,39] revealed that the hydrogen bonding between the protonated N_δ_ on His171 and the *tert*-butoxy ether oxygen of BI-D is unique to the inhibitor because LEDGF/IBD does not similarly contact IN. To examine LEDGF/IBD interactions with the mutant IN we simulated the H171T change in the available crystal structure [20], which revealed that unlike BI-D, LEDGF/IBD made the same number of hydrogen bonds with WT and H171T mutant INs (Additional file 1: Figure S3). In particular, LEDGF/p75 D366 can hydrogen bond with the side chain of Thr171 replacing the lost electrostatic interaction that occurred with His171. To further test these observations, we performed relative binding free energy calculations for LEDGF/IBD binding to WT and H171T IN CCDs. Free energy perturbation analysis allowed us to calculate that *ΔΔ*G_b_ = *ΔG*_*b*_(H171T) − *ΔG*_*b*_(WT) = 1.08 kcal/mol. Since *K*_*d*_ = *e*^*ΔGb*/*RT*^, $\frac{K_d(H171T)}{K_d(WT)}={e}^{\left[\varDelta {G}_b(H171T)-\varDelta {G}_b(WT)\right]/RT}={e}^{\varDelta \varDelta {G}_b/RT}$, the calculated ΔΔG_b_of 1.08 kcal/mol translates into a relative binding affinity ratio $\frac{K_d(H171T)}{K_d(WT)}=6.2$. Experimentally, the *K*_*d*_ of LEDGF/p75 binding to wild type and H171T mutant INs are ~3.3 nM and 10.5 nM respectively, i.e. $\frac{K_d(H171T)}{K_d(WT)}=3.2$, which is in good agreement with the calculated relative affinity ratio of 6.2.

## Discussion

Published studies have shown that ALLINIs are anchored to the IN dimer interface through their key pharmacophore, the carboxylic acid, hydrogen bonding with the backbone amides of IN residues Glu170 and His171. Furthermore, the Thr174 side chain has been implicated in hydrogen bonding with the ALLINI *tert*-butoxy ether oxygen [37,38,40]. However, the contributions of the His171 side chain for interacting with this class of inhibitors have not been previously elucidated. Yet, genotyping of HIV-1_NL4-3_ variants under increasing selective pressure of ALLINI BI-D has revealed the H171T IN substitution as a key amino acid substitution. In addition, this variant persists at the highest inhibitor concentration tested suggesting that the amino acid side chain change at position 171 contributes to the evolved resistance to BI-D [52]. The present studies provide mechanistic and structural clues for these observations. We show that HIV-1_NL4-3_ containing the H171T IN substitution confers ~68-fold resistance to BI-D. Significantly, this level of resistance in infected cells correlates closely with ~84-fold reduced binding affinity of the inhibitor to recombinant H171T IN CCD as compared with its WT counterpart. Crystallographic experiments and binding free energy calculations have indicated that N_δ_-protonated or doubly protonated forms of the imidazole ring of His171 can engage in both electrostatic interactions with BI-D carboxylic acid as well as hydrogen bonding with the *tert*-butoxy ether oxygen of the inhibitor. These interactions are compromised by the H171T substitution, with the Thr171 side chain forming a less electrostatically favorable hydrogen bond with the BI-D carboxylic acid and lacking any additional interactions with the *tert*-butoxy ether oxygen (Figure 5).

In contrast with the significant reduction in BI-D binding affinity, the H171T IN substitution only minimally reduced LEDGF/p75 binding affinity to recombinant H171T IN. In infected cells, where endogenous LEDGF/p75 levels significantly exceed what is needed for HIV-1 integration, HIV-1_NL4-3_ with the H171T substitution was not compromised for HIV-1 replication. The MD simulations and binding free energy calculation have revealed important differences between BI-D and LEDGF/IBD for their binding to HIV-1 IN. The N_δ_ protonated His171 hydrogen bonds the *tert*-butoxy ether oxygen of BI-D, which is compromised upon the H171T IN substitution. In contrast, such interactions are not formed between WT IN and LEDGF/IBD. Accordingly, the H171T IN change minimally affects the IN-LEDGF/p75 binding. Furthermore, unlike BI-D which engages only a small pocket at the CCD-CCD dimer interface, LEDGF/p75 establishes additional extensive interactions with HIV-1 IN, which extend beyond the CCD-CCD dimer interface and include strong electrostatic interactions between positively charged residues along LEDGF/IBD α-helix 4 and a number of acidic residues of α-helix 1 of IN [26]. HIV-1 seems to exploit these structural differences between BI-D and LEDGF/p75 interactions with IN during the process of evolution of the H171T IN escape mutation.

The mechanism for H171T IN resistance is distinct from the previously described mechanism of resistance for the A128T IN escape mutation under the selective pressure of related, archetypal inhibitor BI-1001 [38]. The A128T IN substitution does not significantly reduce BI-1001 binding to HIV-1 IN CCD with all hydrogen bonding and electrostatic interactions of BI-1001 with HIV-1 IN being fully preserved in WT and A128T INs. Instead, the substitution of Ala with bulkier and polar Thr repositioned BI-1001 at the IN CCD dimer interface and reduced its ability to effectively bridge between two IN subunits. Consequently, bound BI-1001 failed to induce aberrant multimerization of recombinant A128T IN and accordingly HIV-1_NL4-3_ containing the A128T IN substitution exhibited marked resistance to BI-1001 [38]. In contrast, the H171T IN substitution was able to resist BI-D through decreasing the ability of the inhibitor to bind IN. However, at high BI-D concentrations, BI-D is able to bind, effectively bridging two IN subunits, and inducing aberrant IN multimerization. Collectively these findings provide important structural and mechanistic details for a novel mechanism of resistance.

Analysis of our crystal structure of BI-D bound to HIV-1 IN CCDs has revealed 13 residues (Gln95, Tyr99, Leu102, Thr124, Thr125, Trp132, Ala128, Ala129, Ala169, Glu170, His171, Lys173 and Met178) that are within 5 Å of the inhibitor. Of these only IN amino acids 124 and 125 are polymorphic with Thr predominating at both positions in clade B, whereas the majority of clade C strains contain Ala124 and Ala125 [55]. Recent studies [33] have shown that these polymorphic substitutions only modestly affected the antiviral potencies of various ALLINIs. We also note close structural similarity between BI-D and BI-224436, the first ALLINI to advance into phase 1a clinical trials ([33], also see Additional file 1: Figure S3). These two compounds exhibit similar antiviral activities in cell culture with an EC_50_ range of 51-90 nM for BI-D and 11-27 nM for BI-224436 with respect to different viral strains [33,39,56]. However, the latter compound has been chosen for clinical trials due to its excellent pharmacokinetic profile in rats [33]. These two compounds differ only in the substituted ring system, with BI-D and BI-224436 containing bicyclic and tricyclic arenes, respectively ([33], also see Additional file 1: Figure S3). While the crystal structure for BI-224436 bound to HIV-1 IN CCDs has not been published, based on its close structural similarity with BI-D we predict that both bi- and tricyclic arenes would similarly dock in the hydrophobic pocket that encompasses Leu102, Ala128, Ala129, Trp132 and Met178. At the same time the key interactions between the N_δ_- protonated imidazole group of His171 with the *tert*-butoxy ether oxygen and electrostatic interactions with the inhibitor carboxylic acid are likely to be equally important for both BI-D and BI-224436 binding to HIV-1 IN. Therefore, our findings are expected to facilitate in the development of improved IN inhibitors for potential clinical use.

## Conclusions

We have elucidated a distinct mechanism of resistance for the H171T IN mutation to the potent ALLINI BI-D. Our findings indicate the importance of the His171 side chain for binding the inhibitor and that its substitution to Thr171 markedly reduces the binding affinity and corresponding inhibitory activity of BI-D.

## Methods

### Antiviral compounds, plasmids, and DNA constructs

BI-D and LEDGIN-6 were synthesized as previously described [37,53]. Plasmid pNL4.3/Xmal [57] encodes for replication competent HIV-1_NL4-3_. Plasmids pNLX.Luc.R^-^ [58] or pNL4-3.Luc.Env- [59] encodes for single-round HIV-1_NL4-3_ carrying the luciferase reporter gene. Vesicular stomatitis virus G glycoprotein was encoded by pCG-VSV-G [57]. The H171T substitution was introduced into the IN coding region of pNLX.Luc.R-, pNL4-3.Luc.Env- and pNL4-3/Xmal using PCR-site directed mutagenesis (Agilent) and verified by dideoxy sequencing.

### Cells, viruses and antiviral assays

Parental HEK293T and HEK293T LEDGF/p75 KO cells [60] were grown in Dulbecco’s modified Eagle medium (Invitrogen) supplemented with 10% (vol/vol) fetal bovine serum (FBS) (Invitrogen), 100 IU/mL penicillin, and 100 μg/mL streptomycin (Gibco). SupT1 cells were maintained in RPMI medium 1640 containing 10% FBS, 100 IU/mL penicillin and 100 μg/mL streptomycin.

HIV-Luc was pseudotyped by cotransfecting HEK293T cells with either pNLX.Luc.R- or pNL4-3.Luc.Env- and with pCG-VSV-G using PolyJet DNA transfection reagent (SignaGen Laboratories).

To determine antiviral activity during virus production, DMSO or the inhibitor was added at indicated concentrations during media exchange at 18-20 hrs post-transfection. Cell-free supernatants were measured for p24 content utilizing a commercial p24 ELISA kit (Advanced Biosciences Laboratories). SupT1 cells were infected in triplicate with HIV-Luc normalized for p24 content (5 ng/mL p24). Luciferase values were determined 48 hrs post-infection.

To assess antiviral activity during the early stage of HIV-1 replication, DMSO or the inhibitor was added at the indicated concentrations to the target cells 30 min before or at the time of infection. Luciferase values, expressed as relative light units were determined 48 hrs post-infection.

### Electron microscopy

Cell-free HIV-1_NL4-3_ from transfected HEK293T cell supernatants was concentrated via ultracentrifugation at 4°C for 2 hrs in a Beckman SW41 rotor at 32,000 rpm prior to fixation in 2% paraformaldehyde and submission to the Harvard Medical School Electron Microscopy core facility. Images were taken with a JEOL 1200EX microscope equipped with an AMT 2 k charge-coupled device camera. Virus particles (100 per experimental sample) were counted by eye.

### Expression and purification of recombinant proteins

WT HIV-1 IN, H171T HIV-1 IN and LEDGF/p75 recombinant proteins with 6xHis or FLAG tags were expressed in *E. coli* and purified as described previously [37]. WT and H171T HIV-1 IN CCD (residues 50-212) containing the F185K mutation were expressed in *E. coli* and purified as described [37].

### Crystallization and X-ray crystal structure determination

Recombinant H171T IN CCD (50-212) containing the F185K solubilizing substitution was prepared to ~8 mg/ml and grown at 4°C using hanging drop vapor diffusion method. The crystallization buffer contains 8% PEG 8 K, 0.1 M Na cacodylate, pH 6.5, 0.1 M ammonium sulfate and 5 mM DTT. Protein (1 μl) was mixed with the equal volume of the crystallization buffer. Within 4 weeks the cubic shape crystals reached 0.1- 0.2 mm in size. The soaking buffer containing 5 mM BI-D was prepared by dissolving the compound in crystallization buffer supplemented with 10% DMSO. The protein crystal was soaked in the buffer for 12 hrs at 4°C before it was flash-frozen with liquid nitrogen. Diffraction data from the crystals were collected at 100 F on a Rigaku Raxis 4++ image plate detector in OSU Crystallography Facility. The intensity data integration and reduction were performed with HKL2000 program [61]. Molecular Replacement program Phaser [62] in CCP4 package method was used to solve the structure. Coot [63] was used for the subsequent refinement and building of the structure. Refmac5 [64] of the CCP4 package was used for the restraint refinement. The crystal belong to space group *P3121* with cell dimensions of *a = b* =72.2 Å, *c* =66 Å; One 18 kDa monomer resides in the asymmetric unit. The structure was refined to 1.94 Å with *Rcryst/R*_free_ =0.190/0.221. Coordinates have been deposited in the Protein Data Bank with accession number 4TSX (Additional file 1: Table S2).

### Binding free energy calculations

The absolute binding free energies between the various forms of IN CCD dimer and BI-D were calculated using the double decoupling method (DDM) [65-68] in explicit solvent (TIP3P water model [69] plus counterions) at 300 K. The protein molecules are modeled by the Amber ff99sb-ILDN force field [70], and the ligand BI-D is described by the Amber GAFF [71] parameters set. The partial charges of the ligands are obtained using the AM1-BCC method [72]. A DDM calculation involves two legs of simulation, in which a restrained ligand is gradually decoupled from the receptor binding pocket or from the aqueous solution. In each leg of the decoupling simulations, the Coulomb interaction is turned off first using 11 lambda windows, and the Lennard-Jones interactions are then turned off in 17 lambda windows. The two decoupling free energies ΔG^gas*→complex^ and ΔG^gas→water^ associated with the two legs of the DDM cycle were determined using thermodynamic integration (TI). The Hamiltonian derivative 〈∂*U*/∂*λ*〉_*λ*_ at a series of l from 0 to 1 were collected and integrated to obtain the free energy difference. For absolute binding free energy calculations, the MD simulation at each λ was performed using the GROMACS [73,74] version 4.6.4 for 15 ns; the last 10 ns was used for the calculation of binding free energy.

### SEC

A Superdex 200 10/300 GL column (GE Healthcare) was used to analyze multimeric forms of recombinant WT and H171T INs in buffer containing 20 mM HEPES (pH 6.8), 750 mM NaCl, 10 mM MgSO_4_, 0.2 mM EDTA, 5 mM BME, 5% glycerol and 200 μM ZnCl_2_. The following proteins were used to calibrate the column: bovine thyroglobulin (670,000 Da), bovine gamma-globulin (158,000 Da), chicken ovalbumin (44,000 Da), horse myoglobin (17,000 Da) and vitamin B12 (1,350 Da). Proteins were detected by absorbance at 280 nm. All the procedures were performed at 4°C.

### DLS

DLS experiments were carried out as previously reported [44]. Briefly, DMSO, 0.12 μM BI-D, or 10 μM BI-D was incubated with 200 nM WT or H171T IN. DLS signals were recorded at room temperature after 15, 20 and 30 minutes using a Malvern Nano series Zetasizer instrument.

### SPR

SPR was performed using a Biacore T100 (GE Healthcare). A series s sensor chip NTA (GE Healthcare) was conditioned with 0.5 mM NiCl_2_ at a flow rate of 10 μl/min for 1 min followed by a 1 min wash of 3 mM EDTA at a flow rate of 10 μl/min. WT or H171T 6xHis-CCD 4 μg/mL in HBS-P, (GE Healthcare) was immobilized on the chip to 4400 response units (RU). Indicated BI-D concentrations in HBS-P with 10% DMSO was flowed over the cell for 180 s at a flow rate of 40 μl/min followed by a 5 min dissociation. The chip was regenerated with 500 mM imidazole.

### Catalytic activities of recombinant INs

LEDGF/p75 dependent integration and LEDGF/p75 independent integration assays were performed using HTRF-based assays as described [38] with minor modifications. LEDGF/p75 independent assays were executed by incubating 400 nM WT or H171T IN with 50 nM Cy-5 labeled donor DNA and 10 nM biotinylated target DNA. For LEDGF/p75 dependent integration assays, 100 nM WT or H171T IN was incubated with 50 nM Cy-5 labeled donor, 10 nM biotinylated target DNA and 50 nM or 100 nM LEDGF/p75. After the addition of europium-streptavidin, the HTRF signal was recorded using a Perkin Elmer EnSpire multimode plate reader.

### HTRF-based assay for IN-LEDGF/p75 interactions

A previously described HTRF-based assay [37] was modified to monitor LEDGF/p75 binding to WT and H171T INs. Briefly, C-terminally FLAG-tagged LEDGF/p75 (0.01 – 100 nM) was titrated in the binding buffer (25 mM Tris, pH 7.4, 150 mM NaCl, 2 mM MgCl_2_, 0.1% Nonidet P-40, 1 mg/ml BSA) containing 10 nM N-terminally 6XHis WT or H171T HIV-1 IN. Anti-6xHis-XL665 and anti-FLAG-EuCryptate antibodies (Cisbio, Inc., Bedford, MA) were added to the reaction and the HTRF signal vs LEDGF/p75 concentration curves were fitted to Hill Equation to identify the *K*_*d*_ values for IN-LEDGF/p75 binding.

## Additional file

Additional file 1:
**Structural analysis of the free energy simulations of BI-D binding to HIV-1 IN CCD;** **Table S1.** Heavy RMS deviation between the simulated bound ligand and the crystal structures; **Table S2.** Data collection and structure refinement statistics; **Figure S1.** Chemical structures of BI-D, LEDGIN-6 and BI-224436; **Figure S2.** The MD simulated structures obtained using different His171 protonation states; **Figure S3.** The MD simulations reveal similar hydrogen bonding between LEDGF/p75 Asp366 with the WT (left) and the H171T mutant (right) IN CCDs.
