# Supplementary material for: The mechanism of H171T resistance reveals the importance of Nδ-protonated His171 for the binding of allosteric inhibitor BI-D to HIV-1 integrase
Source: Retrovirology. 2014 Nov 25;11:100. doi: 10.1186/s12977-014-0100-1 (PMC4251946; doi:10.1186/s12977-014-0100-1)
Supplement: Additional file 1: — Structural analysis of the free energy simulations of BI-D binding to HIV-1 IN CCD; Table S1. Heavy RMS deviation between the simulated bound ligand and the crystal structures; Table S2. Data collection and structure refinement statistics; Figure S1. Chemical structures of BI-D, LEDGIN-6 and BI-224436; Figure S2. The MD simulated structures obtained using different His171 protonation states; Figure S3. The MD simulations reveal similar hydrogen bonding between LEDGF/p75 Asp366 with the WT (left) and the H171T mutant (right) IN CCDs. [file 12977_2014_100_MOESM1_ESM.pdf]

Supplemental Information.

Structural analysis of the free energy simulations of BI-D binding to HIV-1 IN CCD

We have analyzed the MD simulated structures obtained at the fully coupled ensemble (λ = 1) in the binding free energy simulations. It is observed that the bound ligand in the simulated structures maintains the binding mode seen in the crystal structure. The largest RMS deviation from the crystal structure is only 0.62 Å for the ligand heavy atoms (see Supplemental Table 1). The MD simulated structures obtained using different His171 protonation states show important differences in the orientations of the His171 side chain (see Supplemental Figure 1). In both the doubly-protonated His171 and N_δ-protonated His171, the imidazole ring of His171 maintains the orientation seen in the crystal structure which enables it to form the hydrogen bond with the ether oxygen seen in the *tert*-butoxy group of BI-D. In the N_ε-protonated His171 however, the imidazole ring is flipped ~180 degree from the orientation in the crystal structure, and the N_ε-H bond is pointing towards the solvent. No hydrogen bond is formed between the imidazole ring and the ether oxygen. This observation is consistent with the binding free energy calculation reported in Table 2 of the main text, which shows weak ligand-protein interaction in the case of N_ε-protonated His171 receptor.

Supplemental Table 1. Heavy RMS deviation between the simulated bound ligand and the crystal structures. Unit: Å.

Receptor	RMSD
His171-doubly-protonated	0.53
His171-N_δ-protonated	0.57
His171-N_ε-protonated	0.50
The H171T mutant	0.62

Supplemental Table 2. Data collection and structure refinement statistics.

**Data collection**	
Wavelength (Å)	1.541
Unique reflection	15111
Space group	P3121
Unit-cell parameters (Å º)	a=72.248, b=72.248, c=66.032<br>α = β= 90, γ =120
Molecules per ASU	1
Resolution range (Å)	1.94
Completeness (%)	99.9 (100)
Redundancy	4.9 (4.8)
I /σ	48.4 (4.22)
R_{merge} (%)	5.4(44.5)
**Structure refinement**	
Resolution (Å)	1.94
R_{cryst} / R_{free} (%)	0.190/0.221
R.m.s.d from ideal values	
Bond length (Å)	0.0165
Bond angle (º)	1.9265
Average B factor	46
PDB #	4TSX

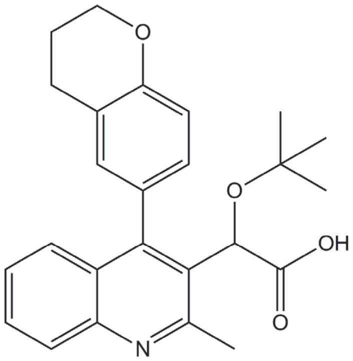

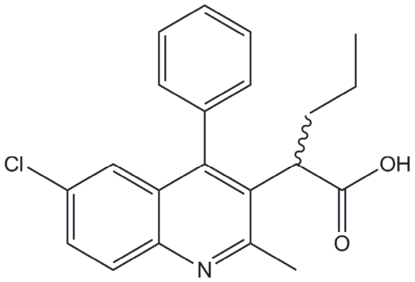

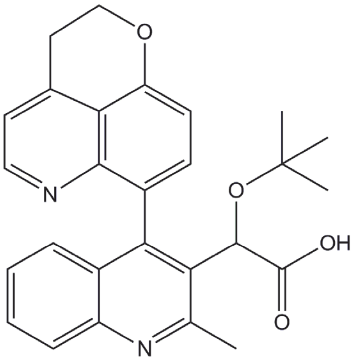

BI-D **LEDGIN-6** **BI-224436**

Supplemental Figure 1. Chemical structures of BI-D, LEDGIN-6 and BI-224436.

Nδ-protonated His171

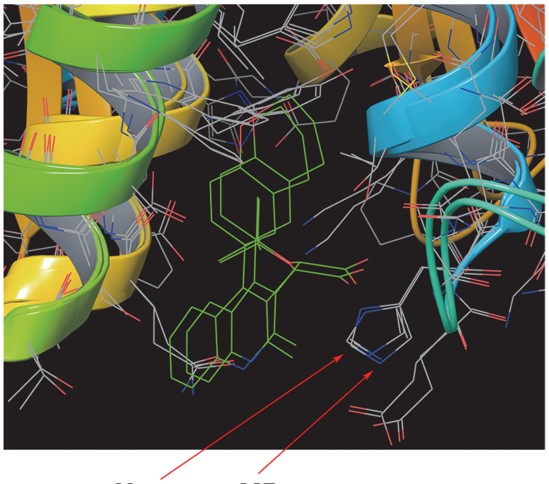

Doubly charged His171

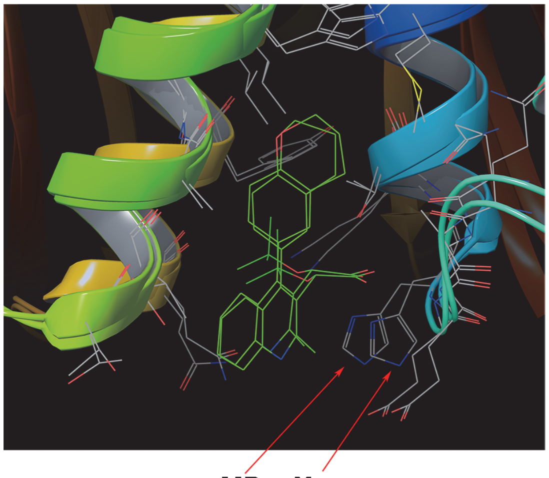

Nε-protonated His171

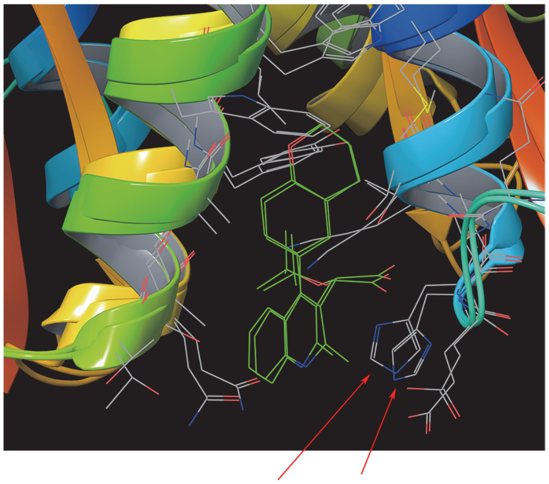

Supplemental Figure 2. The MD simulated structures obtained using different His171 protonation states. The simulated structure at 15 ns is superimposed onto the X-ray crystal structure. The His171 side chain is indicated by the red arrows for the MD simulation (MD) and the crystal structure (X-ray).

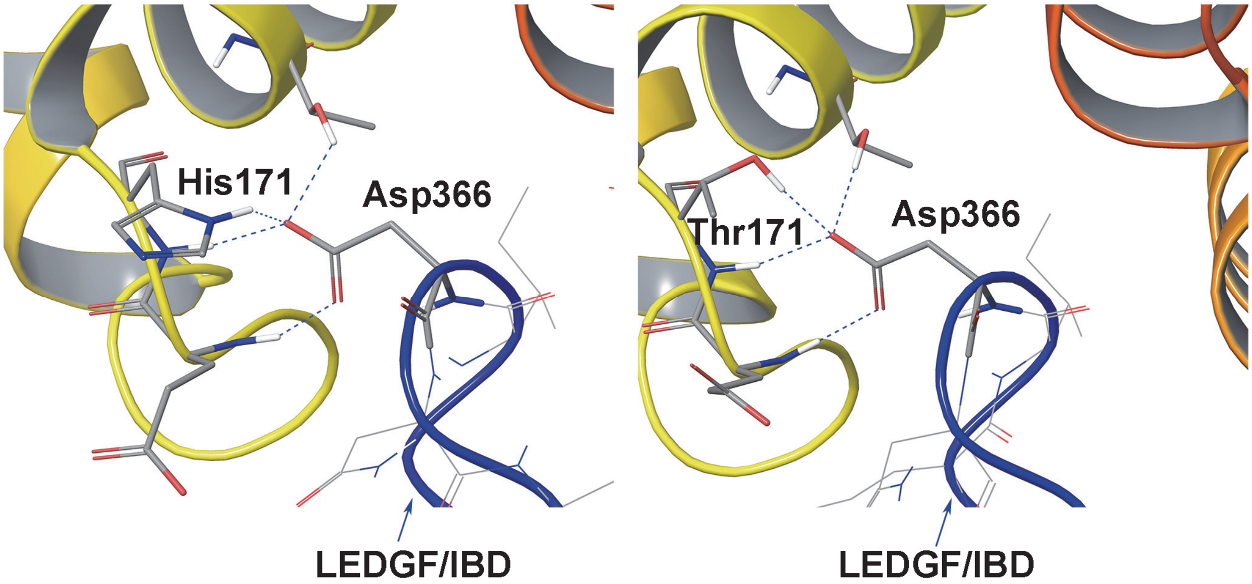

Supplemental Figure 3. The MD simulations reveal similar hydrogen bonding between LEDGF/p75 Asp366 with the WT (left) and the H171T mutant (right) IN CCDs.
